# Yellow Wine Polyphenolic Compounds prevents Doxorubicin‐induced cardiotoxicity through activation of the Nrf2 signalling pathway

**DOI:** 10.1111/jcmm.14466

**Published:** 2019-06-21

**Authors:** Hui Lin, Jie Zhang, Tingjuan Ni, Na Lin, Liping Meng, Feidan Gao, Hangqi Luo, Xiatian Liu, Jufang Chi, Hangyuan Guo

**Affiliations:** ^1^ Department of Cardiology Shaoxing People’s Hospital (Shaoxing Hospital, Zhejiang University School of Medicine) Shaoxing China; ^2^ The First Clinical Medical College Wenzhou Medical University Wenzhou China; ^3^ Zhejiang University School of Medicine Hangzhou China; ^4^ Zhejiang Chinese Medical University Hangzhou China; ^5^ Department of Ultrasound Shaoxing People’s Hospital (Shaoxing Hospital, Zhejiang University School of Medicine) Shaoxing China

**Keywords:** cardiotoxicity, doxorubicin, Nrf2, oxidative stress, polyphenols

## Abstract

Doxorubicin (DOX) is considered as the major culprit in chemotherapy‐induced cardiotoxicity. Yellow wine polyphenolic compounds (YWPC), which are full of polyphenols, have beneficial effects on cardiovascular disease. However, their role in DOX‐induced cardiotoxicity is poorly understood. Due to their antioxidant property, we have been suggested that YWPC could prevent DOX‐induced cardiotoxicity. In this study, we found that YWPC treatment (30 mg/kg/day) significantly improved DOX‐induced cardiac hypertrophy and cardiac dysfunction. YWPC alleviated DOX‐induced increase in oxidative stress levels, reduction in endogenous antioxidant enzyme activities and inflammatory response. Besides, administration of YWPC could prevent DOX‐induced mitochondria‐mediated cardiac apoptosis. Mechanistically, we found that YWPC attenuated DOX‐induced reactive oxygen species (ROS) and down‐regulation of transforming growth factor beta 1 (TGF‐β1)/smad3 pathway by promoting nuclear factor (erythroid‐derived 2)‐like 2 (Nrf2) nucleus translocation in cultured H9C2 cardiomyocytes. Additionally, YWPC against DOX‐induced TGF‐β1 up‐regulation were abolished by Nrf2 knockdown. Further studies revealed that YWPC could inhibit DOX‐induced cardiac fibrosis through inhibiting TGF‐β/smad3‐mediated ECM synthesis. Collectively, our results revealed that YWPC might be effective in mitigating DOX‐induced cardiotoxicity by Nrf2‐dependent down‐regulation of the TGF‐β/smad3 pathway.

## 1 INTRODUCTION

Doxorubicin (DOX), an effective anthracycline, is a chemotherapeutic drug commonly used to treat a wide range of cancers. However, its use is limited due to the potential risk of cardiotoxicity.^1^ Increased mortality associated with the total accumulated dose of DOX has been shown in chemotherapy cycles.^2^ Under this circumstance, researchers have considered designing DOX analogues with reduced acute toxicity; however, this has been confounded by a paralleled loss of anti‐cancer efficacy.^3^ On the contrary, cardioprotective adjuvants, such as dexrazoxane^4^ and vitamin C,^5^ were co‐administered to boost chemotherapeutic efficacy with the purpose of suppressing cardiotoxicity.^6^ Nevertheless, limited evidence supports the cardioprotective effects of these natural compounds and synthetic drugs against DOX‐induced cardiotoxicity.^7^, ^8^

There has been growing interest in the possible benefits of polyphenols due to their antioxidant as well as anti‐apoptotic properties.^9^ Emerging evidence has demonstrated that polyphenols can prevent DOX‐induced cardiotoxicity by exerting their anti‐cancer and cardioprotective effects.^10^ We previously investigated the beneficial effects of yellow wine, which is full of polyphenols, and found that yellow wine polyphenolic compounds (YWPC) could delay the development of atherosclerosis.^11^, ^12^ However, whether YWPC inhibit DOX‐induced cardiotoxicity remains unclear.

To date, multiple molecular mechanisms have been indicated in DOX‐induced cardiotoxicity, and oxidative stress is believed to play a central role,^1^, ^13^ as witnessed by reactive oxygen species (ROS)‐induced injury, including lipid peroxidation and reduced levels of endogenous antioxidant defences.^14^ Oxidative stress also induced activation of several pro‐fibrogenic factors to the subsequent extracellular matrix (ECM) accumulation, leading to the development of cardiac fibrosis.^15^ Therefore, understanding how the oxidant‐antioxidant balance is related to the control of oxidative stress may provide insights into effective therapies for DOX‐induced cytotoxicity. To control oxidative homoeostasis, cells are equipped with an efficient defence system against oxidants. A plenty of studies have identified that nuclear factor‐E2‐related factor‐2 (Nrf2) is implicated as a master regulator in the cellular response to oxidative stress.^16^ Moreover, emerging evidence suggests the importance of inflammation in the pathogenesis of DOX‐induced cardiotoxicity^17^, ^18^ and Nrf2 also has an anti‐inflammatory effect.^19^ Although several studies have revealed that the deactivation of Nrf2 signalling was involved in DOX‐induced cardiotoxicity,^17^, ^20^ whether Nrf2 activation could help to alleviate DOX‐induced cardiac fibrosis are rarely studied.^21^

In this study, we hypothesized that YWPC could ameliorate DOX‐induced cardiotoxicity by inhibiting oxidative stress‐induced cell apoptosis and oxidative stress‐mediated ECM accumulation. To reveal the mechanisms underlying the action of YWPC, we also examined whether YWPC could suppress DOX‐induced oxidative stress through the regulation of Nrf2 signalling pathway.

## 2 MATERIALS AND METHODS

### 2.1 Animals and treatments

**Figure 1:**
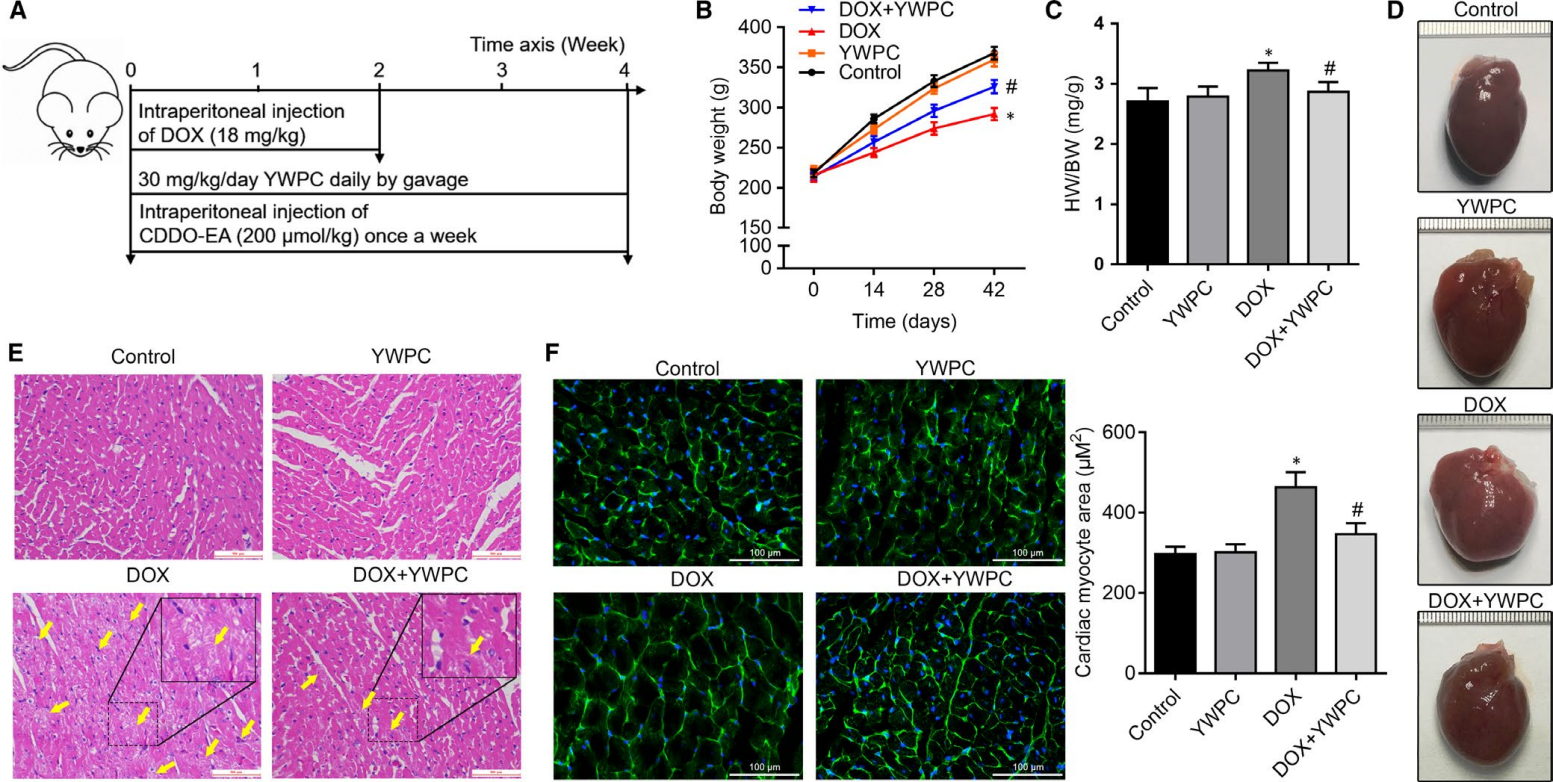
Effect of YWPC on cardiac injury and hypertrophy in rats subjected to DOX intoxication. (A) The study flow diagram for animal experiments. (B) Average body weight change in 4 wk in rats from control group, YWPC group (30 mg/kg/day), DOX groups (18 mg/kg) and DOX + YWPC group (n = 10 per group). (C) Results of heart weight/body weight (HW/BW) in the four groups at fourth week (n = 10). (D) Representative images of global appearance of the heart in different groups. (E) Representative photomicrographs of the heart tissue sections from rats treated with DOX and/or YWPC (H&E staining). The yellow arrow indicates the injured myocardial cell. Bar = 100 μm. (E) WGA staining of left ventricular tissue from rats described in A for determination of cardiac and cardiac myocyte hypertrophy (Bar = 100 μm, n = 10). *P < 0.05, vs. the control; ^#^ *P* < 0.05 vs. the DOX group. CDDO‐EA, CDDO ethyl amide; DOX, doxorubicin; YWPC, Yellow Wine Polyphenolic Compounds

All procedures in this study were performed according to the Guide for the Care and Use of Laboratory Animals from the National Institutes of Health and approved by the Animal Care and Use Committee of Shaoxing People's Hospital. Fifty male Sprague‐Dawley rats (40‐50 days, 185‐210 g), obtained from the Experimental Animal Center of Basic Medicine, Zhejiang Chinese Medical University, were maintained under a 14‐hour light/8‐hour dark cycle at 24°C with free access to rat chow and water. They were randomized into five groups (n = 10 per group): DOX group received intraperitoneal injection of DOX (Cat.HY‐15142A, MedChemExpress, 3 mg/kg, three times per week) for 2 weeks to achieve an accumulative total dose of 18 mg/kg^22^, ^23^; YWPC group received intraperitoneal injection of normal saline and intragastrical injection of YWPC (2 mL, 30 mg/kg/day^12^, ^24^ daily for 4 weeks); DOX + YWPC group received intraperitoneal injection of DOX and simultaneously received YWPC by gavage; DOX + CDDO ethyl amide (CDDO‐EA, Cat.HY‐12213, MedChemExpress) group received DOX and was treated intraperitoneally with CDDO‐EA (200 μmol/kg) dissolved in vehicle containing DMSO, Cremophor EL (Sigma‐Aldrich) and PBS at a 1:1:8 ratio, once a week for 4 weeks^25^, ^26^; Control group received normal saline instead of DOX and received normal saline by gavage (2 mL/day). At the end of the fourth week, rats were inhaled anaesthetized with isoflurane (RWD Life Science Co. Ltd., Guangdong, China) for cardiac function analysis under a small animal anaesthesia machine (R500, RWD Life Science Co. Ltd.). Detailed protocol is illustrated in Figure 1A.

YWPC in dry powder form from yellow rice wine were provided by National Engineering and Research Center for traditional Chinese medicine (Shanghai, China). The procedures used to prepare and analyse YWPC have been described previously.^12^ According to the Food and Drug Administration (FDA) standard for drinking alcohol at a rate of 12‐24 g per day, drinking 200 mL of Shaoxing rice wine with an alcohol content of 12% per day is appropriate. The polyphenol content of Shaoxing rice wine is about 1 mg/mL, so the daily intake of rice wine polyphenols is about 200 mg. According to 70 kg body weight, the intake of rice wine polyphenols is about 2.86 mg/kg. According to the coefficient of 9.1, the weight of 200‐250 g rat polyphenol intervention medium dose is about 30 mg/kg, which was chosen as the appropriate dose for polyphenol intervention in this study.

### 2.2 Transthoracic echocardiography and electrocardiograph recordings

Cardiac echocardiography was performed with the Philips iE33 system (Philips Medical, Best, Netherlands) equipped with an s5‐1 probe (12‐14 MHz) to measure parameters, including left ventricular (LV) ejection fraction (LVEF), LV fractional shortening (LVFS), LV internal dimension‐systole (LVIDs) and LV internal dimension‐diastole (LVIDd). Heart rate was also recorded through the electrocardiograph signal. Philips QLab 9 post‐processing software was used for data analysis.

### 2.3 Histological assessment of myocardial injury and collagen content

After treatment for 4 weeks, all rats were anaesthetized to remove the heart. The heart was quickly photographed and followed by dissecting into small portions and immediately snap‐frozen in liquid nitrogen until analysis. Part of the heart was fixed in 10% formalin, embedded in paraffin, and sectioned at 5‐μm thickness. Haematoxylin and eosin (H&E) staining was used to evaluate the general morphology of the myocardium. The sections were imaged using the Leica DM3000 biological microscope (Leica, Wetzlar, Germany) at 200× magnification. For collagen analysis, the heart sections underwent Masson's trichrome staining. Collagen deposition was quantified using Image‐ProPlus6 (Media Cybernetics, Rockville) as previously described.^27^

### 2.4 Immunohistochemistry

Sections were stained with primary antibodies against TGF‐β1 (ab92486, 1:200), α‐SMA (ab5694, 1:400) and Collagen I (ab34710, 1:400) (all obtained from Abcam), incubated at 4°C overnight, stained with secondary antibodies at room temperature for 30 minutes. Sections were then washed with PBS and incubated with 3,3'‐Diaminobenzidine (DAB) (Gene Tech, Shanghai, China) for 5 minutes at room temperature. The detailed procedure has been previously described.^28^

### 2.5 Wheat germ agglutinin (WGA) staining

Part of each heart form rats was quickly stored at −80°C. The 5‐μm thick frozen heart sections were fixed with 4% PFA for 15 minutes and washed with PBS for three times. Subsequently, the sections were incubated with 5 μg/mL WGA (L4895; Sigma‐Aldrich) solubilized in PBS for 20 minutes at room temperature in darkness. After washeing three times with PBS, the sections were stained with DAPI (P36941; Invitrogen) for 5 minutes at room temperature in dark. A Nikon Eclipse Ti‐U fluorescence microscope (Minato‐ku, Tokyo, Japan) was used to acquire images and a total of 30 fields per section were observed at 400× magnification and analysed with ImageJ software (National Institutes of Health). The cardiac myocyte size was determined by the measurement of total area divided by the number of cardiac myocytes.

### 2.6 Measurement of oxidative stress and antioxidant enzyme activity

Dihydroethidium (DHE) staining was used for reactive oxygen species (ROS) detection. Fresh frozen sections were incubated with 10 μmol/L of DHE (Beyotime, Jiangsu, China) at 37°C for 30 minutes, followed by incubation with 4′,6‐Diamidino‐2‐phenylindole dihydrochloride (DAPI) for 3 minutes. Images were then acquired by a Nikon Eclipse Ti‐U fluorescence microscope. Lipid Peroxidation MDA Assay Kit (Cat.S0131, Beyotime) was used to measure the levels of lipid peroxidation. Antioxidant enzyme activity was then tested using Catalase Assay Kit (Cat.S0051) and Total Superoxide Dismutase (SOD) Assay Kit (Cat.S0101), all of which were obtained from Beyotime. Experiments were performed in the cardiac tissue in accordance with the manufacturer's instructions.

### 2.7 Measurement of TNF‐β1, IL‐1β and IL‐6

Heart tissues were lysed with protein lysis buffer, and the lysates were subject to determine the levels of pro‐inflammatory cytokines (TNF‐β1, IL‐1β and IL‐6) using ELISA Kits (Qiagen, Inc, Hilden, Germany), according to the manufacturer's instructions. Optical densities were measured using a SpectraMax Plus Absorbance microplate reader (Molecular devices, CA) at 450 nm.

### 2.8 Transmission electron microscopy

A portion of the left ventricle was from each rat cut into 1 mm fragments and was fixed in 2.5% glutaraldehyde overnight for electron microscopic examination. The tissues were fixed in 1% osmic acid followed by dehydrated in a series of acetone washes. The tissues were then embedded in Araldite for coronal sections and sectioned at 1 μm. The slides were stained with toluidine blue and ultra‐thin sections were cut in from this block and were observed under a Cs‐corrected TEM with Monochromator (Titan G2 60‐300, FEI, Hillsboro).

### 2.9 Apoptosis assay

An in situ cell death detection kit, POD (Roche Co. Ltd., Basel, Switzerland), was used to detect DNA fragmentation of individual cells. Paraffin‐embedded sections of the heart tissues were deparaffinized with xylene and rehydrated in a graded ethanol series. The sections were incubated with proteinase K at 37°C for 20 minutes. Tissues then were incubated with the TdT‐mediated dUTP nick end labeling (TUNEL) reaction mixture at 37°C for 1 hour in the dark. Subsequently, the sections were covered with converter‐POD at 37°C for 30 minutes, followed by incubation with DAB for 3 minutes. After counterstaining with the nuclear counterstain hematoxylin for 30 seconds, the number of TUNEL‐positive cells was counted with a Leica microscope at 100× magnification.

### 2.10 Immunofluorescence

Frozen tissue sections were used for immunofluorescence assay to visualize the expression of caspase 3. Briefly, the cryostat sections were thawed at room temperature for 20 minutes and followed by incubation in blocking buffer (4% BSA) for 30 minutes. Subsequently, immunostaining was performed with an anti‐caspase 3 antibody (ab32351, Abcam) and an Alexa Fluor 594 WGA (Life Technologies). Nuclei were counterstained with 0.1 μg/mL DAPI (Sigma‐Aldrich; D9564). Images were captured using a Nikon Eclipse Ti‐U fluorescence microscope at ×400 magnification.

### 2.11 Cell culture and treatment

H9C2 cells were obtained from Cell Bank of Chinese Academy of Sciences (Shanghai, China) and cultured in DMEM (Sigma) supplemented with 10% foetal bovine serum (Gibco, Grand Island, NY), and 1% U/mL penicillin and streptomycin (Beyotime, Jiangsu, China). Cells were maintained at 37°C in a humidified atmosphere of air containing 5% CO_2_. Cells were treatment with 5 μmol/L DOX for 24 hours to establish cell toxic model. To investigate the role of YWPC in vitro, cells were pre‐treatment with 50 mg/L YWPC or 100 μmol/L CDDO‐EA (MedChem Express).^29^ To knockdown of Nrf2 in H9C2 cells, the Nrf2 small interfering RNA (siRNA) (5′‐GUAAGAAGCCAGAUGUUAA‐3′) and control siRNA (5′‐UUCUCCGAACGUGUCACGUTT‐3′) oligonucleotides were synthesized by GenePharma (Shanghai, China). A single dose of 30 nmol of siRNA was administrated to the cells at 60% confluency by transfection with 250 μL of Lipofectamine 3000 (Invitrogen) in Opti‐MEM medium (Gibco, CA) according to the manufacturer's instructions. Knockdown efficiencies were tested by Western blot analysis 48 hours after the siRNA transfection.

### 2.12 Western blot analysis

Total protein was extracted from stored frozen tissues using RIPA lysis buffer (Beyotime) containing phosphatase inhibitor cocktail II (MedChem Express, China), and 30 μg of protein was separated by SDS‐PAGE and transferred to PVDF membranes (Millipore, MA). The membranes were then blocked with 5% skim milk for 1 hour at room temperature and hybridized with primary antibodies against Bcl‐2 (#15071, 1:500), Bax (#2772, 1:1000), cytosolic cytochrome C (#4280, 1:1000), cleaved caspase‐3 (#9664, 1:1000), Nrf2 (ab62352, 1:1000), p‐smad3 (ab52903, 1:1000), smad3(ab40854, 1:1000), LaminB (ab16048, 1:1000) or β‐actin (1:2000) (obtained from Abcam, Cambridge, MA) at 4°C overnight. Subsequently, the membrane was treated with secondary antibody (1:5000; Abbkine, Redlands, CA), and visualized using an ECL detection kit (Cat: 32106, Pierce™ ECL Western Blotting Substrate, Thermo scientific). Quantity One software was used for analysis.^30^

### 2.13 Real‐time reverse‐transcription PCR

Total RNA was isolated from hearts tissues using TRIzol reagent (Invitrogen) following the manufacturer's protocol. RNA was reverse‐transcribed with oligo (dT) primers and real‐time PCR was conducted on an ABI 7300 RT‐PCR Detection System (Applied Biosystems, Foster, CA) with gene‐specific primers in the presence of SYBR Premix Ex Taq (TaKaRa). RT‐qPCR was performed in triplicate. Relative fold changes in the expression of the target gene were determined using the 2^−△△CT^ method.^31^ The primer sequences used were: HO‐1 forward, 5′‐CGGGCCAGCAACAAAGTG‐3′ and reverse, 5′‐CGGGCCAGCAACAAAGTG‐3′; NQO‐1 forward, 5′‐TTCCGGAGTAAGAAGGCAGT‐3′ and reverse, 5′‐GAAGCCACAGAAATGCAGAA‐3′; GCLM forward, 5′‐ATGGAGTTCCCAAATCAGCC‐3′ and reverse, 5′‐ATTGGGTTTTACCTGTGCCC‐3′; GAPDH forward, 5′‐GCACCGTCAAGGCTGAGAAC‐3′ and reverse, 5′‐TGGTGAAGACGCCAGTGGA‐3′.

### 2.14 Assessment of mitochondrial membrane potential

JC‐1 method was used to measure mitochondrial membrane potential as described previously.^32^ In brief, following treatment, H9C2 cells were incubated with 2.5 mmol/L JC‐1 dye (Solarbio, Beijing, China) for 30 min in the dark at 37°C. Red JC‐1 aggregates represented a normal hyperpolarized membrane potential, while Green JC‐1 monomers indicated a loss of mitochondrial membrane potential. Images were obtained by the fluorescence microscope at 400× amplification.

### 2.15 Cell apoptosis assay

After treatment, H9C2 cells were stained with Annexin V‐fluorescein isothiocyanate (FITC) Apoptosis Kit (Beyotime, Jiangsu, China) in accordance with the manufacturer's instructions. Briefly, cells were harvested and washed three times with PBS. Subsequently, cells were resuspended in Annexin V binding buffer and stained with Annexin V‐FITC and propidium iodide in the dark for 15 minutes at room temperature. Stained cells were analysed by flow cytometry (FACS Calibur, Bio‐Rad Laboratories, Inc, USA) within 1 hour.

### 2.16 Statistical analysis

Results are shown as mean ± standard deviation (SD). Differences among groups were analysed using one‐way ANOVA, followed by Tukey's post hoc analysis. Data were analysed with the SPSS version 20.0 software (SPSS Inc, Chicago, IL). *P* < 0.05 was considered statistically significant.

## 3 RESULTS

### 3.1 YWPC improve DOX‐induced cardiac hypertrophy

As shown in Figure 1B, body weight gain was significantly decreased in the DOX group as compared to the control group. However, co‐administration of DOX and YWPC could reverse this trend. At the fourth week, the heart weight/body weight was higher in the DOX group than in the DOX + YWPC and control groups (Figure 1C). The global appearance of the heart in the indicated four groups has been shown in Figure 1D. The rats in the DOX group had an enlarged heart, whereas the remaining rats in the other three groups had similar normal heart. H&E staining of the heart sections of the DOX group demonstrated a great amount of cell injury (karyolysis, disorganization of the muscle fibres and interstitial oedema) compared with the control group. Interestingly, in rats that were co‐treated with YWPC, the normal myocardial architecture was preserved and cell injury significantly reduced (Figure 1E). Further, we found that DOX‐associated enlargement in cardiomyocyte size was attenuated in YWPC‐treated rats as assessed by WGA staining (Figure 1F). Collectively, these results revealed that YWPC exhibit an effective protective role in DOX‐induced cardiac hypertrophy.

### 3.2 YWPC improve DOX‐induced cardiac dysfunction

**Figure 2:**
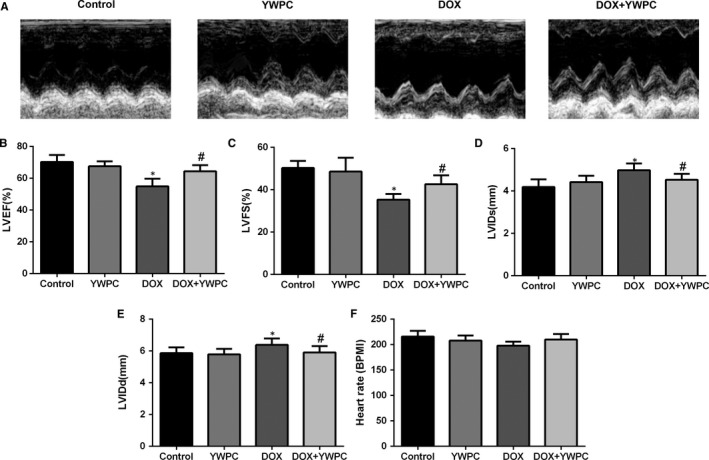
YWPC exert cardioprotective effects on DOX‐induced cardiac dysfunction. (A) Anatomic M‐mode echocardiography and corresponding electrocardiographic images of Sprague‐Dawley rat hearts. Results of (B) LVEF, (C) LVFS, (D) LVIDs, and (E) LVIDd in the four groups (n = 10). (F) Mean Heart rate in different treatment groups (n = 10). *P < 0.05, vs. the control; ^#^ *P* < 0.05 vs. the DOX group. DOX, doxorubicin; LVEF, left ventricular ejection fraction; LVFS, left ventricular fractional shortening; LVIDs, left ventricular internal dimension‐systole; LVIDd, left ventricular internal dimension‐diastole; YWPC, Yellow Wine Polyphenolic Compounds

Echocardiography was then used to monitor the improvement in cardiac function (Figure 2A). Results suggested that LVEF (Figure 2B) and LVFS (Figure 2C) were similar in the YWPC and control groups, but significantly reduced in the DOX group, which was then enhanced by YWPC treatment. Compared to control group, DOX treatment markedly increased LVIDs (Figure 2D) and LVIDd (Figure 2E), which were ameliorated by YWPC treatment. However, the results of electrocardiography showed no significant difference in heart rate between the groups (Figure 2F).

### 3.3 YWPC prevent DOX‐induced oxidative stress, in vivo

**Figure 3:**
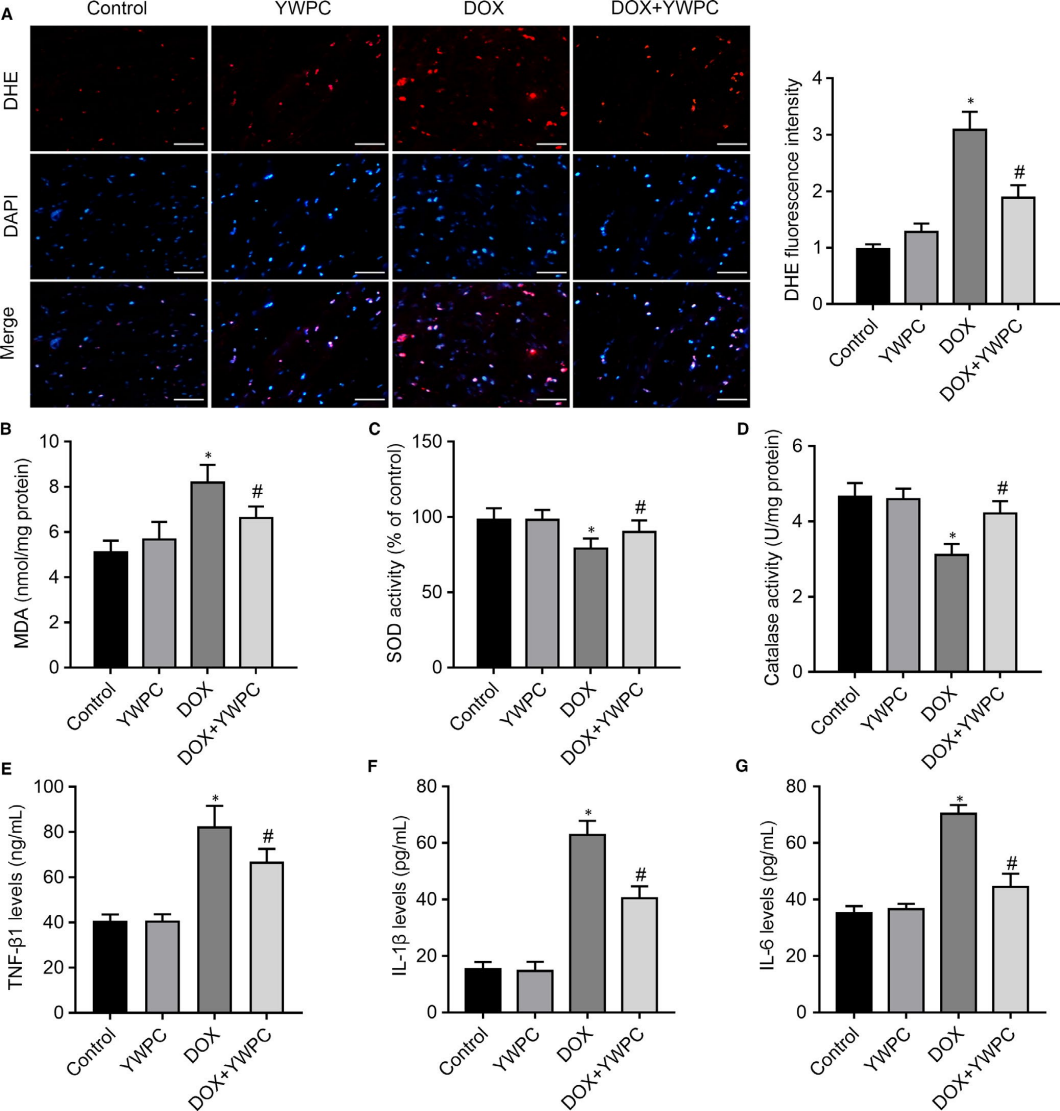
YWPC treatment ameliorates DOX‐induced oxidative stress, in vivo. (A) Staining cells with red indicated DHE‐positive cells; DAPI staining (blue) indicated nucleus. Bar = 50 μm. Percentages of DHE‐positive cells of total cells were shown. Cardiac antioxidant enzyme activities of (B) MDA, (C) SOD and (D) catalase were evaluated using in the heart homogenates from indicated groups. Enzyme‐linked immunosorbent assay (ELISA) was used on serum from rats to determine the expression of transforming growth factor‐β1 (TGF‐β1) (E), interleukin‐1β (IL‐1β) (F), and interleukin‐6 (IL‐6) (G). Data are shown as the mean ± standard deviation of 10 rats. *P < 0.05, vs. the control; ^#^ *P* < 0.05 vs. the DOX group. DOX, doxorubicin; MDA, malondialdehyde; SOD, Superoxide Dismutase; YWPC, Yellow Wine Polyphenolic Compounds

To further understand the molecular mechanisms associated with the beneficial effects of YWPC, the levels of oxidative stress and endogenous antioxidant enzyme activities in the cardiac tissue were measured. As shown in Figure 3A, DHE staining showed increased number of DHE‐positive cells in the DOX group, indicating the generation of ROS. YWPC effectively reduced the DOX‐induced generation of ROS. In addition, high levels of lipid peroxidation production MDA were observed in DOX‐stimulated rats, which were significantly decreased with the treatment of YWPC (Figure 3B). Subsequently, a significant decrease in the activity of antioxidant enzymes, including SOD and catalase was observed in DOX‐treated rats as compared to control and YWPC‐treated rats (Figure 3C and 3D). YWPC intake dramatically alleviated the reduction in antioxidant enzyme activity in the DOX + YWPC group. Furthermore, the levels of pro‐inflammatory cytokines, TNF‐β1, IL‐1β, and IL‐6 were significantly increased in the DOX group compared with the control and YWPC groups; however, their levels were reduced in the DOX + YWPC group compared to the DOX group (Figure 3E‐G). These results indicated that YWPC significantly alleviated DOX‐induced oxidative damage through modulating the balance of oxidation and antioxidation.

### 3.4 YWPC attenuate DOX‐induced myocardial apoptosis

**Figure 4:**
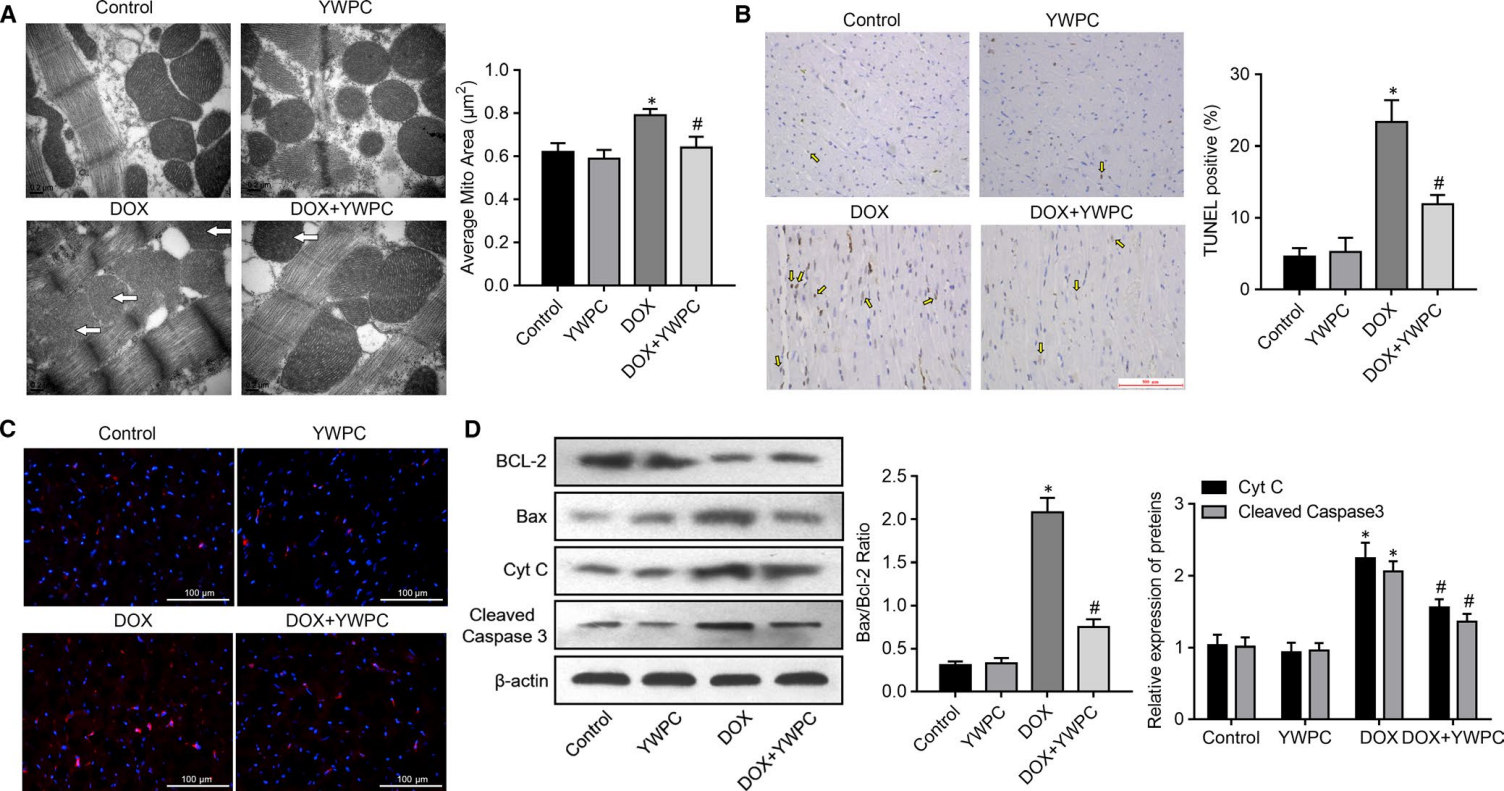
YWPC prevent DOX‐induced cardiac myocyte apoptosis, in vivo. (A) Transmission electron microscopy showing betterment of mitochondrial ultrastructure in DOX + YWPC group compared to DOX‐treated rats. Bar = 0.2 μm, 50 000× magnification. Corresponding graphs showing mitochondrial diameter and average mitochondrial area in vivo from different treatment groups (n = 10). (B) Representative images of TUNEL staining of the heart tissues. Apoptotic cardiomyocyte nuclei appear brown‐stained and normal nuclei appear blue. (C) Caspase‐3 expression in the different treatment groups were detected via immunofluorescence analysis. (D) Protein expression of Bcl‐2, Bax, Cyt C and cleaved caspase‐3 in the different treatment groups. *P < 0.05, vs. the control; ^#^ *P* < 0.05 vs. the DOX. DOX, doxorubicin; YWPC, Yellow Wine Polyphenolic Compounds

**Figure 5:**
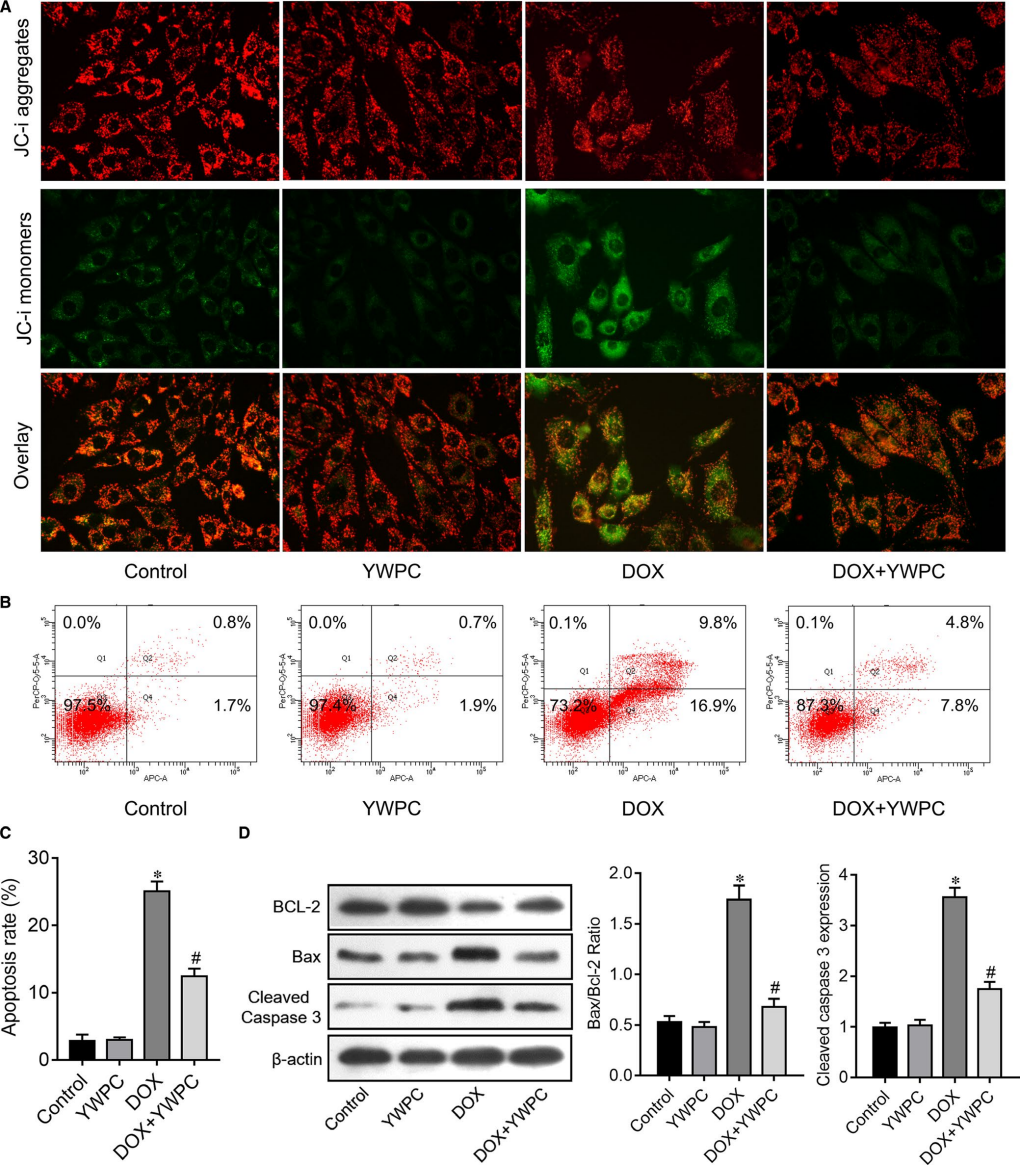
YWPC prevent DOX‐induced cardiac myocyte apoptosis, in vitro. (A) Effects of YWPC (50 mg/L for 24 h) on DOX (5 μmol/L for 24 h)‐induced dissipation of mitochondrial membrane potential measured in H9C2 cells loaded with JC‐1 using fluorescence microscopy. Magnification ×400. (B‐C) Effects of YWPC on DOX‐induced cell apoptosis of H9C2 cells, as detected by Flow cytometry. (D) The relative expression of Bcl‐2, Bax and cleaved caspase‐3 in H9C2 cells with or without YWPC treatment under DOX stimulation. *P < 0.05, vs. the control; ^#^ *P* < 0.05 vs. the DOX. DOX, doxorubicin; YWPC, Yellow Wine Polyphenolic Compounds

Because apoptosis plays a key role in DOX‐associated cardiotoxicity, we thus explored the effects of YWPC on DOX‐induced cell apoptosis. The mitochondria of the cardiomyocytes in the DOX group showed loss of cristae, swelling and distortion, along with significant increase of average mitochondrial area compared to the control group, as revealed by transmission electron microscopic analyses. In comparison, there was well‐integrated ultrastructure in the mitochondria in the DOX + YWPC group compared with the DOX group (Figure 4A). As expected, DOX induced significant increase in myocardial apoptosis as detected by TUNEL staining. YWPC treatment of DOX‐treated rats attenuated apoptosis, compared to the DOX group (Figure 4B). Protein expression of apoptosis‐related proteins (mitochondrial pathway) was then evaluated. The level of cleaved caspase 3 was dramatically increased in the DOX group but decreased in the DOX + YWPC group as detected by immunofluorescence assay (Figure 4C). DOX stimulation increased the ratio of Bax/Bcl‐2 and cytosolic cytochrome c and cleaved caspase‐3 expression levels. However, YWPC treatment reversed above changes significantly (Figure 4D). To validate above effects of YWPC on cell apoptosis, DOX‐induced H9C2 cells were introduced as a cell model in vitro. Mitochondrial membrane potential was measured using the JC‐1 method. The control cells exhibited red JC‐1 aggregates, representing a normal hyperpolarized membrane potential. After DOX (5 μmol/L for 24 hours) incubation, cells exhibited green JC‐1 monomers, representing a loss of mitochondrial membrane potential. However, YWPC treatment displayed light red and orange fluorescence, indicating obvious protection against DOX‐induced mitochondrial membrane potential loss (Figure 5A). In H9C2 cells incubated with DOX for 24 hours, a remarkable increase in cell apoptosis was observed. However, YWPC treatment markedly suppressed DOX‐stimulated cell apoptosis (Figure 5B, C). In addition, consistent with the results in vivo, YWPC treatment notably reduced DOX‐stimulated Bax/Bcl‐2 ratio and cleaved caspase‐3 (Figure 5D). Taken together, these results indicated that YWPC prevent DOX‐induced mitochondria‐mediated cardiac apoptosis.

### 3.5 YWPC attenuate DOX‐induced reactive oxygen species (ROS) and down‐regulation of transforming growth factor beta (TGF‐β)/smad3 pathway dependent on Nrf2 status

**Figure 6:**
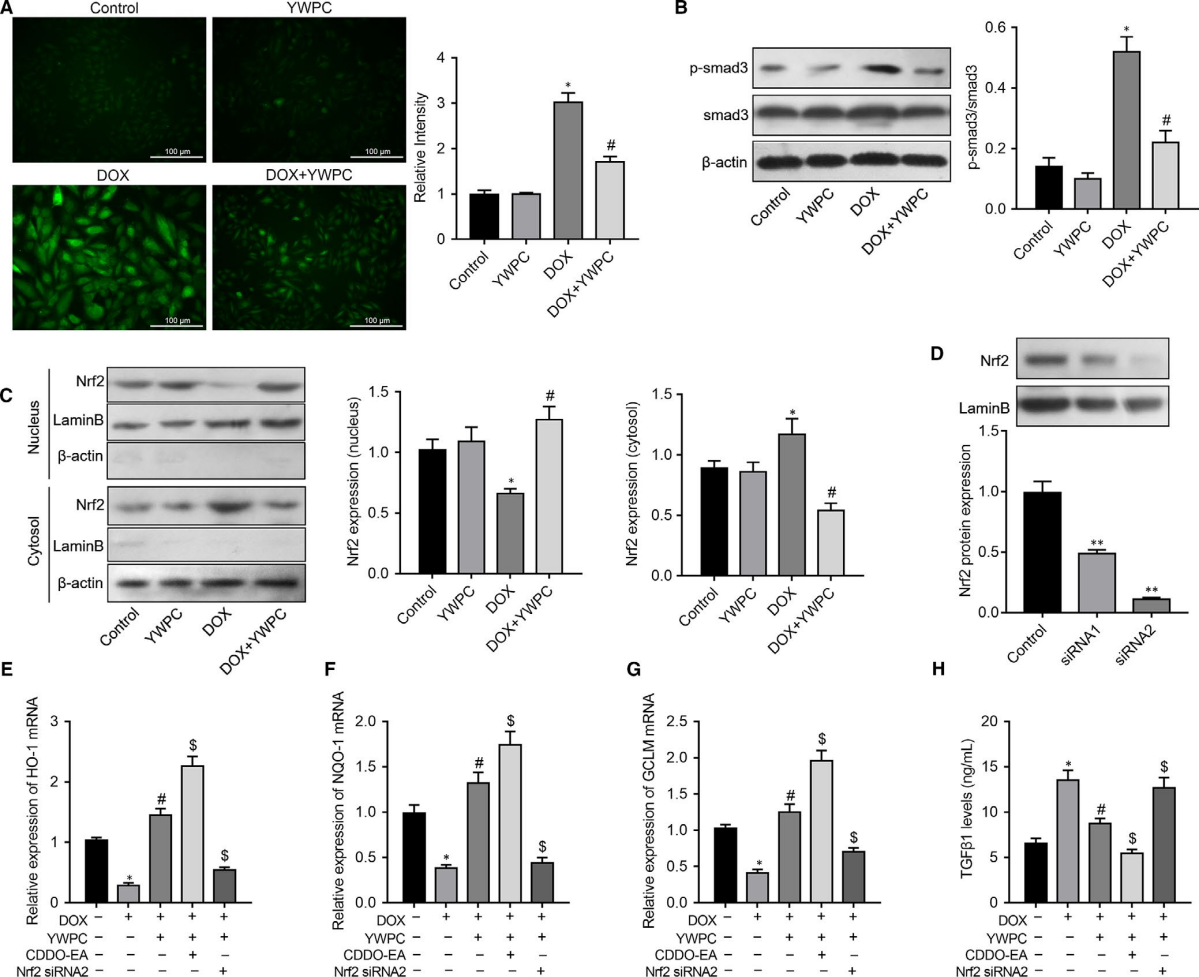
YWPC alleviate DOX‐induced reactive oxygen species (ROS) and down‐regulation of transforming growth factor beta (TGF‐β)/smad3 pathway dependent on Nrf2 status, in vitro. (A) The intracellular ROS production of H9C2 cells under the stimulation of DOX (5 μmol/L for 24 h) with or without pre‐treatment of YWPC (50 mg/L for 24 h) was visualized under a fluorescence microscope with 2′, 7′‐dichlorodihydrofluorescein diacetate (DCFDA) probe. (B) The protein expression level of p‐smad3 and smad3 in H9C2 cells was determined by Western blot using phosphorylated smad3 and smad3 antibodies. (C) DOX treatment for 6 h induced the cytosol accumulation of Nrf2, and YWPC pre‐treatment enhanced the nuclear accumulation of Nrf2 activated by DOX; (D) Knockdown of Nrf2 in H9C2 cells was achieved by specific siRNA and the efficiency validated by Western blot analysis. RT‐qPCR was used to determine the expression levels of Nrf2 target genes, HO‐1 (E), NQO‐1 (F), and GCLM (G) in H9C2 cells managed with DOX and/or YWPC, CDDO‐EA Nrf2 siRNA. (H) The TGF‐β1 expression level in the culture supernatants was analysed by Enzyme‐linked immunosorbent assay (ELISA). *P < 0.05, vs. the control; ^#^ *P* < 0.05 vs. the DOX. DOX, doxorubicin; YWPC, Yellow Wine Polyphenolic Compounds

ROS scavenging is a powerful way to protect against DOX‐induced cardiac damage.^33^ To explore the molecular mechanisms underlying the antioxidant effects of YWPC, we examined the effect of YWPC on the intracellular ROS level of (5 μmol/L for 24 hours) DOX‐stimulated H9C2 cells using DCFDA. As illustrated in Figure 6A, treatment with 50 mg/L YWPC suppressed the intracellular ROS production of H9C2 cells, which triggered by DOX. A large number of studies have manifested the critical role of TGF‐β1/smad3 signalling pathway in cardiac fibrosis. Using antibodies that recognize the unphosphorylated and phosphorylated form of smad3, we found that phosphorylation of smad3 induced by DOX was significantly attenuated in the presence of YWPC (Figure 6B). In addition, due to the critical role of Nrf2 in regulation of oxidative stress, we subsequently explored whether YWPC were involved in the regulation of Nrf2 expression. Intriguing, when compared to the control group, 6 hours of DOX treatment notably up‐regulated the cytosol expression levels of Nrf2, while down‐regulated its nucleus levels. As expected, YWPC treatment obviously promoted Nrf2 translocation from cytoplasm to nucleus (Figure 6C). To confirm the role of ROS and Nrf2 in YWPC‐induced suppression of TGF‐β/smad3 pathway, H9C2 cells were treated with transfected with Nrf2 siRNAs (Figure 6D). Using RT‐qPCR, we identified that treatment of H9C2 cells with DOX resulted in a significant decrease in levels of downstream genes Nrf2, including HO‐1, NQO‐1 and GCLM. However, this effect could be reversed by YWPC treatment. We subsequently found that CDDO‐EA (a Nrf2 activator) could further up‐regulate YWPC‐enhanced antioxidant genes, while knockdown of Nrf2 could abolish YWPC‐enhanced antioxidant genes (Figure 6E‐G). Importantly, we found that YWPC reduced the DOX‐induced TGF‐β1 levels in the culture supernatants of H9C2 cells. CDDO‐EA treatment further decreased TGF‐β1 levels while Nrf2 silencing increased TGF‐β1 levels (Figure 6H). In summary, these data indicated that YWPC attenuate DOX‐induced ROS via TGF‐β1/smad3 pathway in a Nrf2 dependent manner.

### 3.6 YWPC alleviate DOX‐induced cardiac fibrosis, in vivo

**Figure 7:**
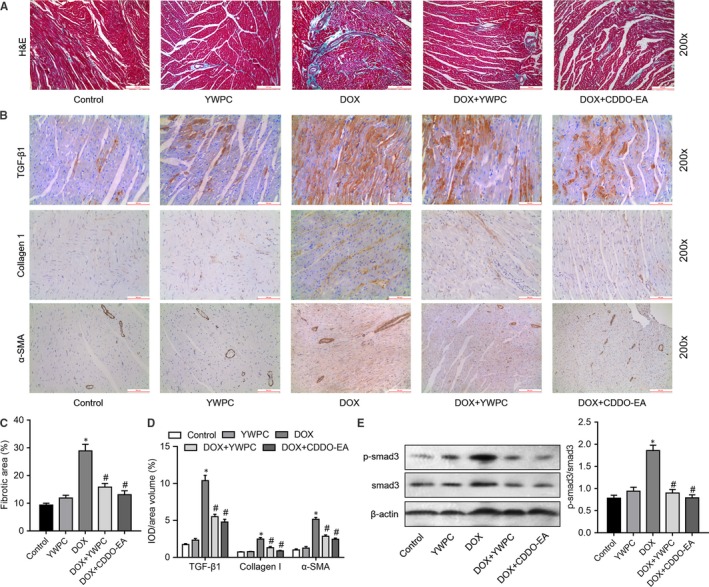
YWPC suppress DOX‐induced cardiac fibrosis by inhibiting TGF‐β1/smad3 pathway, in vivo. (A) Masson's trichome staining was used to evaluate collagen deposition in the heart tissue from control group, YWPC group, DOX group, DOX + YWPC group and DOX + CDDO‐EA group. (B) Relative expression of TGF‐β1, Collagen I and α‐SMA were determined by immunohistochemical staining. (C) Collagen volume fraction was quantified (positive area/total area) in groups. (D) Relative expression of TGF‐β1, Collagen I and α‐SMA protein in myocardial tissue were quantified of the results in B. (E) The protein expression of p‐smad3 and smad3 in myocardial tissue was determined by Western blot. *P < 0.05, vs. the control; ^#^ *P* < 0.05 vs. the DOX. CDDO‐EA, CDDO ethyl amide; DOX, doxorubicin; YWPC, Yellow Wine Polyphenolic Compounds

**Figure 8:**
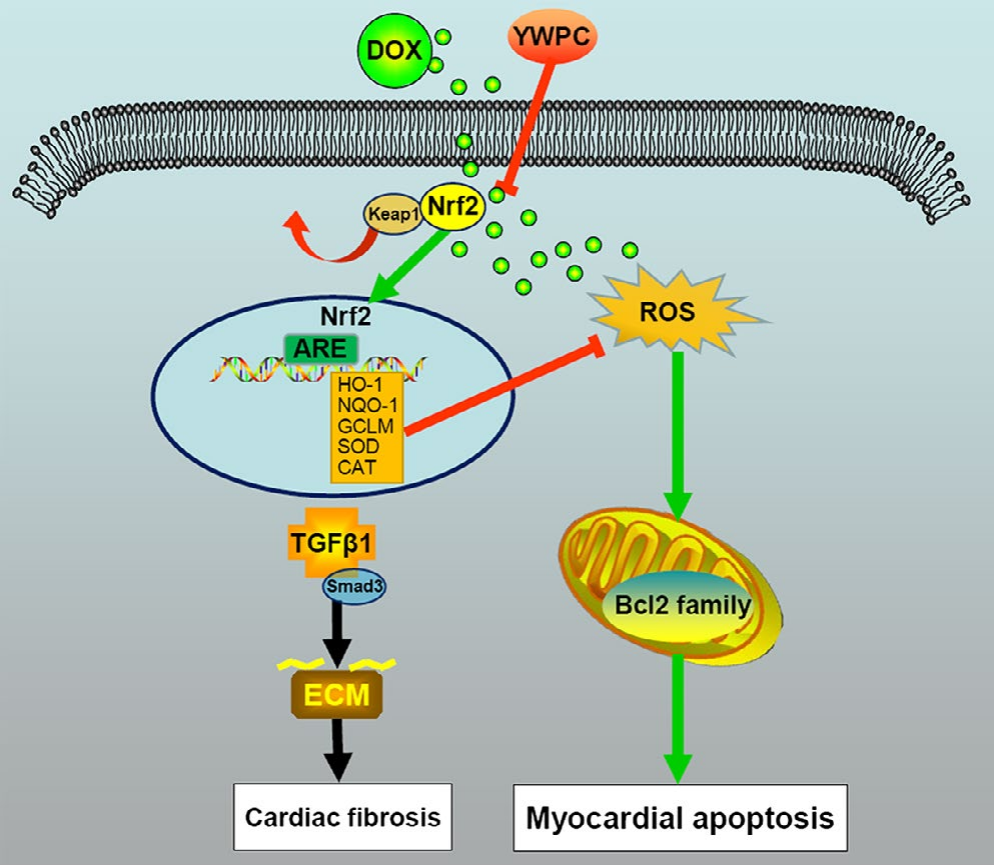
Schematic diagram showing the cardioprotective effects of YWPC on DOX‐induced cardiotoxicity. DOX, doxorubicin; YWPC, Yellow Wine Polyphenolic Compounds

To validate the anti‐fibrosis effects of YWPC, we then measured the changes of fibrotic proteins in DOX‐injected rats with or without YWPC or CDDO‐EA treatment. As shown in Figure 7A, C, the results of Masson's trichrome staining showed that compared with control group, the collagen content in the heart tissues of DOX‐treated rats was increased, which was then significantly reduced by YWPC or CDDO‐EA treatment. In addition, the pro‐fibrotic factor TGF‐β1, the ECM content collagen I and the cardiac fibrosis‐associated protein α‐SMA were remarkably increased in DOX‐treated rats compared to those in control group, as detected by IHC. However, the levels of TGF‐β1, Collagen I and α‐SMA were reduced by the addition of YWPC or CDDO‐EA (Figure 7B, D). Western blot analysis then showed that DOX significantly increased p‐smad3 expression, which was reduced by YWPC or CDDO‐EA treatment (Figure 7E). Above Generally, Figure 8 showed the protective effects and potential mechanisms of YWPC on DOX‐induced cardiotoxicity.

## 4 DISCUSSION

In this study, we demonstrated that YWPC effectively alleviated DOX‐induced cardiotoxicity by inhibiting oxidative stress through activation of Nrf2 pathway. The cardioprotective effects of YWPC were validated through several lines of evidence. First, YWPC could improve the reduction of cardiac function and cardiac hypertrophy induced by DOX. Second, YWPC treatment dramatically reduced the ECM accumulation and inhibited DOX‐induced cardiac fibrosis. Mechanically, we found that YWPC significantly attenuated DOX‐triggered oxidative stress and increased the levels of antioxidant enzyme activities. In addition, we demonstrated that the YWPC inhibited DOX‐induced mitochondria‐dependent apoptosis. Moreover, we identified that YWPC significantly reduced DOX‐induced TGF‐β1/smad3 pathway through up‐regulated Nrf2 nuclear translocation. Inhibition of these pathways might be an important reason for the cardioprotective effects of YWPC on DOX‐induced cardiotoxicity.

YWPC used in our study were derived from Shaoxing rice wine and have been identified owning the cardiovascular protective effects through inhibiting matrix metalloproteinase‐2, ‐9 expression during atherogenesis.^11^, ^12^, ^34^ The main mechanisms by which polyphenols are cardioprotective are related to the improvement of vascular function and their anti‐atherogenic effect.^35^ Of note, the use of polyphenols is currently being investigated as a possible adjunctive therapy in combination with DOX to protect the myocardium from DOX‐induced oxidative stress.^36^ DOX accumulates in the mitochondria and disrupts the mitochondria electron chain resulting in an enhanced production of ROS.^37^ This enhanced production of reactive radicals can react with proteins, lipids and DNA molecules, causing DNA damage and lipid peroxidation.^38^, ^39^ Oxidative stress through increased generation of ROS and compromised antioxidant defence are well documented in DOX‐related myocardial apoptosis.^5^, ^40^ Consistent with previous studies,^41^ we found DOX treatment markedly induced ROS generation and reduced the activity of endogenous antioxidant enzymes, such as SOD and catalase in the heart tissues. This DOX‐induced oxidative stress in the heart could be attenuated by 30 mg/kg YWPC treatment, which may be largely due to the antioxidant properties of polyphenols.^7^ Currently, there are plenty of polyphenol monomers identified have the cardioprotective effects of DOX‐induced cardiotoxicity because of their antioxidative effects. For instance, resveratrol has the ability to ameliorate DOX‐induced myocardial structure damage and cardiac dysfunction in rats through inhibiting free radical generation, improving mitochondrial dysfunction and suppressing apoptosis.^42^ Epigallocatechin‐3‐gallate possesses cardioprotective action against DOX by suppressing oxidative stress and inflammation, as well as activation of pro‐survival pathway.^43^ Consistently, we identified that YWPC mitigated cellular damage by reducing ROS generation, improving antioxidant enzyme activities, resulting in inhibition of DOX‐induced mitochondria‐mediated myocardial apoptosis in DOX‐treated rats and cardiac cells. However, the potential mechanisms underlying the action of YWPC remain to be elucidated.

Excessive ROS generation has been identified as an important pathogenic factor of DOX‐induced cardiotoxicity. DOX‐derived ROS causes an imbalance between pro‐ and anti‐apoptotic proteins (such as Bcl‐2 family^44^), disrupting mitochondrial membrane potential, causing cytochrome c release and subsequently causing cell apoptosis.^45^ The heart has high energy requirement and a high mitochondrial density, which is more susceptible to DOX‐induced toxicity.^46^ With the treatment of YWPC, we found great improvement in the disordered and swelled mitochondria induced by DOX. Researchers have confirmed that DOX treatment affects mitochondrial gene expression and suppresses cardiac mitochondrial metabolism and biogenesis, resulting in mitochondrial dependent apoptosis.^47^ Here, we found that YWPC reduced the level of DOX‐induced mitochondria‐mediated apoptosis of cardiac myocytes, with the up‐regulation of Bcl‐2 and down‐regulation of Bax, and release of cytochrome C and cleaved caspase‐3.

Cardiac fibrosis is the common feature of many cardiac pathophysiological conditions, and has been considered to be involved in cardiac stiffness and dysfunction in DOX‐induced cardiotoxicity.^21^ In the present study, we found that co‐administration with YWPC caused a remarkable inhibition of TGF‐β1/smad3 pathway, reduction of ECM and mitigatory fibrosis in DOX‐treated rats. TGF‐β1/smad3 pathway is considered to be the most potent and ubiquitous pro‐fibrogenic cytokines that involved in the development of fibrosis.^15^, ^48^ It also reported that DOX triggered fibrosis through α‐SMA, Collagen I and III regulation. Consistently, we found the protein levels of Collagen I and α‐SMA were dramatically increased in DOX‐treated rats, indicating cardiac fibroblasts activation and excessive ECM deposition. After YWPC treatment, ECM proteins were reduced. A rapidly expanding body of evidence supports that Nrf2 overexpression alleviates ROS accumulation and oxidative damage by mediating TGF‐β1/Smad3 signalling inhibition.^49^, ^50^, ^51^ Once exposure to ROS, Nrf2 dissociates from Keap1 and translocates into the nucleus to activate the transcription of various cytoprotective genes including HO‐1, NQO‐1 and GCLM.^52^ Because Nrf2 exerts its cytoprotective effects against oxidative stress after translocation into the nucleus from the cytoplasm,^53^ we examined the effects of YWPC on the expression of Nrf2 in H9C2 cells exposed to DOX. Suppression of Nrf2 signalling pathway was observed after 24 hours’ DOX stimulation, indicating DOX induced more reactive oxidants production than antioxidants, causing net oxidative stress in DOX‐treated cardiac myocytes. Our results demonstrated that treatment with YWPC could activate the Nrf2 pathway to prevent oxidative injury in DOX‐treated H9C2 cells. Our results further showed that knockdown of Nrf2 could reverse the inhibitory effects of YWPC on DOX‐induced TGF‐β1 levels. A recent study revealed that siRNA‐mediated knockdown of Nrf2 could down‐regulate Tanshinone IIA‐induced Nrf2 activation and reverse the effect of Tanshinone IIA on the DOX‐induced suppression of cell viability.^53^ Furthermore, Li et al identified that knockout of Nrf2 could exaggerate DOX‐induced cardiotoxicity and cardiac dysfunction by controlling oxidative stress and autophagy.^54^ On the contrary, using COOD‐EA (a Nrf2 inducer) as a positive control, our study confirmed that YWPC could active Nrf2 pathway in DOX‐induced rats to inhibit the progress of cardiac fibrosis, in vivo. Taken together, our data supported the idea that YWPC could activate Nrf2 signalling pathway and attenuate the DOX‐induced toxicity.

Although it is still unsure whether YWPC have the ability to reinforce the chemotherapeutic efficacy of DOX or have anti‐cancer effects, the current study found that YWPC supplementation alleviated DOX‐induced cardiac dysfunction, as well as migrated cardiac fibrosis, and discussed its potential mechanisms. However, our study had certain limitations. Firstly, YWPC used in this experiment are a mixture of different monomer components, and which one of them plays a protective role is still unclear. Besides, findings based on DOX‐induced cardiotoxicity in SD rats could not fully represent the situations and pathology in human; thus, further validations in clinical study are needed in future study.

Collectively, our data showed that YWPC supplementation protects against oxidative stress, mitochondrial damage, fibrosis, hypertrophy and apoptosis, eventually improving cardiac function in DOX‐treated rats. The cardioprotective role of YWPC is contributing from its ability to inhibit DOX‐stimulated oxidative stress by augmenting the Nrf2 pathway. The results suggested that YWPC might be a promising novel therapeutic strategy to reduce DOX‐induced cardiotoxicity.

## CONFLICT OF INTEREST

The authors declare that the research was conducted in the absence of any commercial or financial relationships that could be construed as a potential conflict of interest.

## AUTHOR CONTRIBUTIONS

JFC and HYG designed the project; HL, JZ, TJN, NL and XTL performed animal experiments; LPM and FDG performed in vitro experiments; HL, LPM and FDG analysed the data; HYG and JFC supervised and funded the project; HL and LPM wrote the draft manuscript and HYG made the modification.

## Data Availability

The data used to support the findings of this study are included in the article.
